# Experimental Evidence of the Viability of Thermoelectric Generators to Power Volcanic Monitoring Stations

**DOI:** 10.3390/s20174839

**Published:** 2020-08-27

**Authors:** Leyre Catalan, Amaia Garacochea, Alvaro Casi, Miguel Araiz, Patricia Aranguren, David Astrain

**Affiliations:** Department of Engineering, Institute of Smart Cities, Public University of Navarre, 31006 Pamplona, Spain; amaia.garacochea@unavarra.es (A.G.); alvaro.casi@unavarra.es (A.C.); miguel.araiz@unavarra.es (M.A.); patricia.arangureng@unavarra.es (P.A.); david.astrain@unavarra.es (D.A.)

**Keywords:** thermoelectric generator, volcano surveillance, power supply, geothermal, LoRa, autonomous, heat pipe

## Abstract

Although there is an important lack of commercial thermoelectric applications mainly due to their low efficiency, there exist some cases in which thermoelectric generators are the best option thanks to their well-known advantages, such as reliability, lack of maintenance and scalability. In this sense, the present paper develops a novel thermoelectric application in order to supply power to volcanic monitoring stations, making them completely autonomous. These stations become indispensable in any volcano since they are able to predict eruptions. Nevertheless, they present energy supply difficulties due to the absence of power grid, the remote access, and the climatology. As a solution, this work has designed a new integral system composed of thermoelectric generators with high efficiency heat exchangers, and its associated electronics, developed thanks to Internet of Things (IoT) technologies. Thus, the heat emitted from volcanic fumaroles is transformed directly into electricity with thermoelectric generators with passive heat exchangers based on phase change, leading to a continuous generation without moving parts that powers different sensors, the information of which is emitted via LoRa. The viability of the solution has been demonstrated both at the laboratory and at a real volcano, Teide (Canary Islands, Spain), where a compact prototype has been installed in an 82 ${}^{{}^{\circ}}$C fumarole. The results obtained during more than eight months of operation prove the robustness and durability of the developed generator, which has been in operation without maintenance and under several kinds of meteorological conditions, leading to an average generation of 0.49 W and a continuous emission over more than 14 km.

## 1. Introduction

Volcanoes are considered one of the most dangerous natural hazards [1]. Nowadays, more than 10% of the global population lives within 100 km of a volcano that has the potential to erupt [2]. Therefore, volcanic vigilance becomes indispensable in any volcanic system of the world, so that the damage caused by the inevitable eruptions can be reduced.

Normally, volcanic eruptions are preceded by anomalous signals, known as precursors [3,4]. Volcanic vigilance consists in the monitoring of these signals in order to, together with knowledge of past activity, predict when, where, and how the next volcanic eruption will occur. For this purpose, due to the diverse nature of the different manifestations of volcanic activity, volcano surveillance requires the combination of different techniques, the data of which needs to be analyzed together. Seismology studies the earthquakes and other generating events of seismic waves [5]; geodesy focuses on the variations in the shape and dimensions of the Earth [6]; geochemistry studies the change in temperature and composition of the volcanic products [7]; gravimetry deals with variations of the gravitational forces [8]; and magnetotellurics analyzes the fluctuations in the electromagnetic fields of the Earth [9].

In order to obtain data, most of the previous techniques use local measurements. There exist some remote methodologies such as GPS, GNSS, InSAR or satellite temperature monitoring [10,11]. Nevertheless, local measurements are preferred due to their better accuracy and the coverage of all techniques [12]. Among local measurements, two trends can be in turn distinguished: continuous and discontinuous data acquisition [13]. In the former, permanent vigilance stations are installed in key locations, while the latter consists in performing in situ manual measurements. In general, if possible, continuous monitoring is more recommended, due to its lower cost (does not require several people taking measurements) and more information provided. Nevertheless, power supply and communication systems of such stations constitute a challenge [12], since volcanic areas are usually remote, inaccessible, and lack a power grid. This causes that only 30% of active volcanoes have instrumentation to measure their activity [14].

In order to face the previous situation, the most common alternative consists in using photovoltaic panels and, consequently, batteries, so that power supply is ensured during nights, periods of absence of sun, or episodes of volcanic ashes [15,16]. However, sometimes, depending on the climatology and the specific conditions, the required capacity of the batteries is so high that it is preferred to lose data during some periods, rather than deal with the installation difficulties of an enormous battery in a remote and inaccessible location. This is the case of those volcanoes that record severe snowfalls during long periods in winter such as Mt. Fuji in Japan [17], or that are located at extreme latitudes where the sun does not shine for months, like Deception Island in Antarctica [18].

In the last years, with the purpose of reducing the capacity of the batteries, low-energy devices have proliferated, focusing on both data acquisition and communications. Hence, it stands out the use of systems with embedded Linux, which permit real-time monitoring with a power consumption lower than 2.5 W [16,18,19]. These systems are usually combined with communication technologies normally associated with Internet of Things (IoT). For instance, Awadallah et al. developed a wireless network of thermometers to measure soil temperatures in volcanic areas based on LoRa [20]. LoRaWAN protocol was also chosen by Terray et al., but in this case to control soil radon emissions [21].

Apart from reducing the consumption of the different elements that compose a vigilance station, another solution consists in using an alternative energy source, such as geothermal energy, which presents the advantage of being always available independently of weather. In active volcanoes, the most evident manifestation of geothermal energy coincides with one of the signs of active volcanism: fumaroles, vents in the Earth’s surface from which steam and volcanic gases are emitted, normally at temperatures between 70 and 100 ${}^{{}^{\circ}}C$ [22]. Hence, this solution fits in the proverb “*if you cannot beat them, join them*”.

Traditional geothermal cycles are not suitable for low-scale power generation from volcanic fumaroles. Nonetheless, thermoelectric generators could become an alternative. Thermoelectric generators are devices based on solid state physics whereby heat (i.e., temperature difference) is directly transformed into electricity by means of the Seebeck effect. Thanks to these devices, the operation of which is more deeply described in Section 2, it is possible to generate electricity continuously from geothermal heat [23,24], even improving with adverse meteorological conditions, thus permitting a reduction in the capacity of the batteries or the utilization of other storage technologies such as supercapacitors. Furthermore, these devices are modular, compact, and have demonstrated their reliability and durability without maintenance requirements in spatial applications [25].

Considering the former advantages, the use of thermoelectric generators have already been proposed to power ground sensors. Due to the difference in heat capacity and conduction rate between the air and the ground, there normally exists a temperature difference between them [26]. Lawrence [27] and Stevens [28] demonstrated that this temperature difference can be transformed into electricity by means of thermoelectric generators, permitting the power supply of remote sensors. As indicated by Stokes et al., this type of self-sufficient power source could serve for unattended ground sensors (UGS) in defense and security [29]. They experimented with this proposal at the laboratory, developing a complete sensor consisting of a thermoelectric generator, a DC-DC boost converter, a wireless sensor transmitter, and supercapacitors, which presented good performance with temperature differences as low as 1 ${}^{{}^{\circ}}C$.

Forest monitoring is another possible application. Similarly to volcano surveillance, forests are also monitored in order to analyze the impact of droughts or fires, to uncover their structure, and to study variations of their biota, for which wireless sensors are used [30,31,32]. Nevertheless, these sensors also present energy supply problems due to their remoteness. As a solution, Wang et al. proposed the utilization of thermoelectric generators to provide a stable power supply, taking advantage of the mentioned temperature difference between the ground and the air [33]. Huang et al. improved the previous micro-generator and performed field experiments under natural conditions in two different locations [34]. The results obtained over six months reveal that the location influences the power generation that can be obtained. Hence, in Harbin an average of 0.335 mW were generated, while in Beijing, only 0.076 mW could be produced. In a later article, they concluded that in order to efficiently harvest micro-energy from shallow soil, the thermoelectric generator needs to be placed where the soil moisture is greater than 30% [35].

Roadway and bridge infrastructures also require energy harvesting in order to power a multitude of data collection and communication applications [36]. For this purpose, it is considered that thermoelectric generators are one of the most readily available methods [37], transforming the heat absorbed from the exposure to solar radiation into electricity. For instance, Tahami et al. fabricated a system to embed into asphalt pavements, so that with the temperature difference between the pavement surface and the soil below it, electricity could be generated. They optimized and tested a prototype in the field, obtaining 29 mW [38]. Another alternative consists in making use of the temperature difference between road surface and ambient air, as proposed by Jiang et al., who obtained up to 45 mW [39].

Thermoelectric generators can also represent a solution for the power supply of volcanic monitoring stations. In comparison with the previous examples, this application presents the advantage of having a heat source with higher temperature, which is beneficial for the efficiency improvement of thermoelectric generators. In contrast, the device needs to operate in a harsher environment and requires to be more robust, being able to work without maintenance, for which moving parts should be avoided.

The objective of the present paper is to develop an autonomous and robust volcanic monitoring station powered by thermoelectric generators. For this purpose, Section 2 details the operation of thermoelectric generators in their application to volcanic fumaroles; Section 3 describes the thermoelectric generator that has been developed; Section 4 characterizes the previous generator at the laboratory; Section 5 details the electronics of the device, also analyzing alternative communication systems typically associated with Internet of Things (IoT); Section 6 examines the behavior of the complete system in a real volcano; Section 7 studies supplying power to a real vigilance station; and finally Section 8 summarizes the main contributions of the present paper.

## 2. Operation of a Geothermal Thermoelectric Generator (GTEG)

As exposed in the introduction, thermoelectric generators are devices based on solid state physics whereby heat (i.e., temperature difference) is directly transformed into electricity by means of the Seebeck effect. This transformation is held in the so-called thermoelectric modules, the efficiency of which is proportional to the temperature difference between their sides. In order to maximize this temperature difference, the introduction of heat exchangers with low thermal resistances becomes indispensable, so that the temperature of the hot side of the thermoelectric modules approaches to the temperature of the heat source, and the temperature of the cold side does the same with respect the dissipation sink (normally the environment). An 8% higher generation can be obtained with a reduction of 10% in the thermal resistance of the heat exchangers [40]. Fin dissipators, heat exchangers with a fluid as heat carrier, heat pipes, and thermosyphons are the most common heat exchangers used in thermoelectric generators [41].

Thanks to these devices, it is possible to generate electricity directly, avoiding the intermediate conversion of thermal energy into mechanical energy; regardless of the source temperature, only a temperature gradient is required; durably, as demonstrated in spatial applications; with scalability possibilities, increasing the installed power just by adding more thermoelectric modules; and without moving parts, working fluids and noise in the operation of their main element, the thermoelectric modules [42]. As a drawback, they present a very low efficiency, which can be rated between 2 and 5% depending on the temperature range. Nevertheless, as derived from the introduction, there exist some applications in which the benefits of thermoelectric generators counteract their main disadvantage, becoming the best alternative. This is the case of supplying power to volcanic monitoring stations, the application under consideration in the present paper.

In this application, fumaroles represent the heat source. These fumaroles, in their emergence to the surface, heat up and moisten the soil. Hence, due to the low heat transfer coefficient of gases, the thermoelectric generator responsible for the power supply will be directly in contact with the hot and wet soil. For depths higher than 10% it is considered that the temperature of the soil equals the fumaroles’ one and that the soil moisture is close to 90%, thus a good heat transfer from the soil is expected [35].

**Figure 1:**
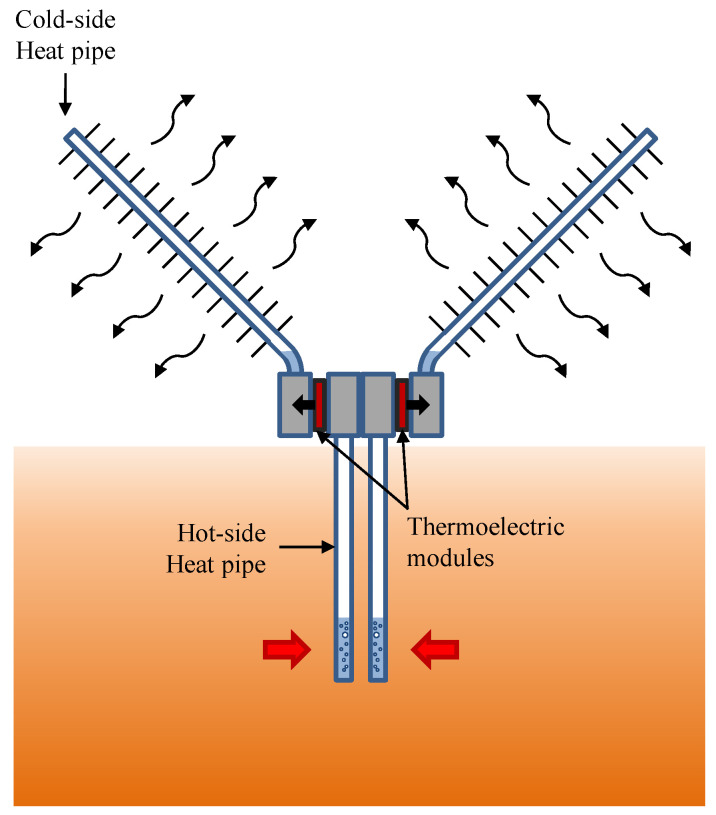
Schematics of a geothermal thermoelectric generator (GTEG) with heat pipes as heat exchangers.

The purpose of the hot side heat exchanger consists of absorbing the volcanic heat underground and transmitting it, with a minimal temperature difference to the thermoelectric modules located overground, so that the hot side of the thermoelectric modules approaches the temperature of the fumaroles. Catalan et al. demonstrated that heat exchangers based on phase change are the most adequate ones for this task [43], and therefore, will be the ones considered in this paper. As depicted in Figure 1, this type of heat exchanger is made of one or several tubes with an internal fluid in their interior. In contact with the heat source, the internal fluid vaporizes, occupying all the available space. Afterward, this vapor condensates releasing heat to the thermoelectric modules. If the condensed fluid returns to the evaporator part only due to gravity, the heat exchanger is known as thermosyphon, while if a wick material is introduced so that it can work in any position, the term heat pipe is used. Lawrence, Wang et al., and Huang et al. also used phase change heat exchangers, specifically heat pipes, to absorb heat from the soil and transfer it to the thermoelectric modules [27,33,34,35].

The heat that is not transformed into electricity by the thermoelectric modules needs to be dissipated into the environment, again with a heat exchanger with a minimal thermal resistance so that the cold side of the thermoelectric modules approach the ambient temperature. In the previous examples [27,33,34,35], fin dissipators in natural convection were used due to their simplicity and low cost. Nonetheless, in this case, heat exchangers based on phase change will be again used since they have demonstrated to be the most suitable ones for the cold side of the modules, leading to low thermal resistances without requiring auxiliary consumption [43,44]. Their mode of operation is similar to the hot side one, with cyclic vaporization and condensation of the internal fluid, although in this case heat is absorbed from the thermoelectric modules and released into the environment (Figure 1). The main difference between them is that in the cold side heat exchangers, in order to improve convection between the exterior part of the tube and the ambient, fins are normally added, so that the heat exchange area is increased.

Altogether, the use of heat exchangers based on phase change leads to a noiseless, compact, robust, and modular generator, with no auxiliary consumption nor moving parts, and therefore without maintenance requirements. Furthermore, since a constant temperature heat source is being used, a continuous power supply is obtained.

## 3. Prototype Description

**Figure 2:**
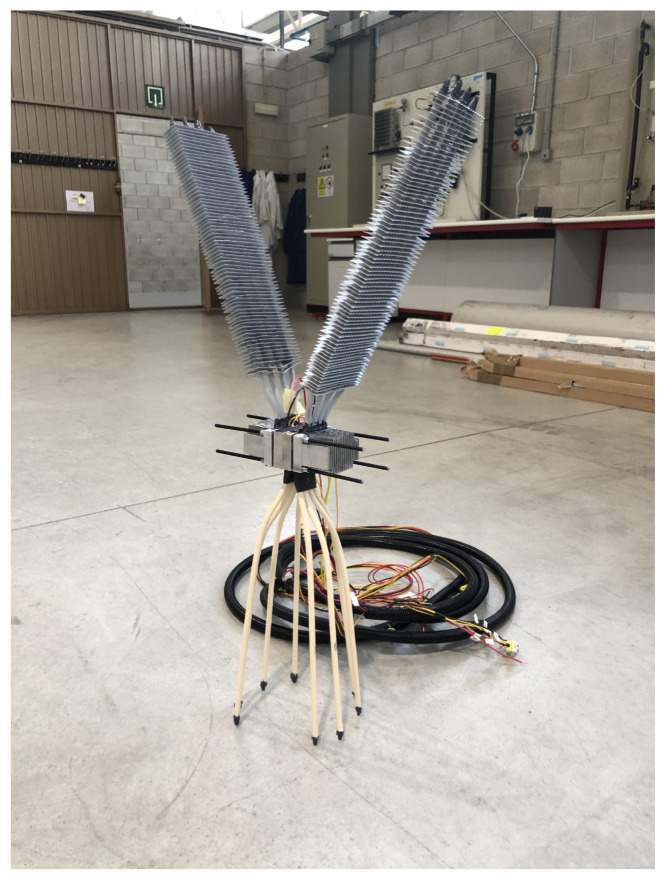
Designed thermoelectric generator composed of two thermoelectric modules and heat pipes as heat exchangers.

Figure 2 depicts the thermoelectric generator developed in the present paper to be installed in volcanic fumaroles and supply power to volcanic monitoring stations. The prototype is composed of two thermoelectric modules, each of them with its own heat exchangers.

The hot side heat exchanger of each module is composed of four 450 mm long copper sintered heat pipes with an external diameter of 8 mm. The tubes have been inserted in an 82 × 50 × 20 mm ${}^{3}$ aluminum plate in which four holes with a slightly smaller diameter than the heat pipes have been drilled with a separation of 3 mm, so that a good thermal contact is ensured.

Each cold side heat exchanger has also been manufactured with the former procedure. Nonetheless, in this case, 500 mm long heat pipes have been inserted in the base of an aluminum fin dissipator. This dissipator has a base of 82 × 50 × 14.5 mm ${}^{3}$ and seventeen 40 × 1.5 mm ${}^{2}$ corrugated fins. Fins have also been inserted on the heat pipe tubes in order to increase the heat exchange area and therefore, improve heat dissipation to the environment. More specifically, 72 aluminum 104 × 27.5 × 0.3 mm ${}^{3}$ fins have been added with a separation of 5 mm.

The hot and cold heat exchangers are assembled by means of four M5 threaded rods, which due to their closeness, ensure a good pressure distribution in the thermoelectric modules and therefore a suitable thermal contact [45]. For compactness, the same threaded rods are used to assembly a generator with two thermoelectric modules, as shown in Figure 2. In this paper, commercial bismuth telluride modules have been used. More specifically, Marlow TG12-8-01LS [46]. These modules are sealed with silicone, so that the thermocouples are protected, something especially important in this application due to the harsh volcanic environment.

The heat exchangers also need to be protected. Hence, the heat pipes of the hot side heat exchanger have been coated with an epoxy-based primer [47], while the cold side ones with a marine primer [48]. Furthermore, the weakest parts, such as the ends of the heat pipes and the inter-phases with the aluminum plates or the fin dissipators, have been protected with an epoxy adhesive [49].

## 4. Laboratory Characterization

This section details the characterization of the previous prototype at the laboratory. First, it describes the characterization of the cold side heat exchanger. Afterward, the complete characterization of the whole thermoelectric generator is developed.

The characterization of the cold side heat exchanger (CHE) refers to the determination of its thermal resistance for different heat fluxes. For this purpose, a 40 × 40 mm ${}^{2}$ heating plate has been used to provide the desired heat flux ${\dot{Q}}_{CHE}$ in each experiment, and the temperature at the base of the heat exchanger $T_{b}$ and in the climatic chamber $T_{amb}$ have been measured. Thanks to these measurements, the thermal resistance has been calculated according to Equation (1). Each experiment has been repeated three times and the uncertainties have been obtained with [50], considering thermocouples with precision $\pm 1\;{}^{{}^{\circ}}C$. Insulation was also added to ensure that all the heat flux went through the heat exchanger.

(1) $$R_{CHE}=\frac{T_{b}-T_{amb}}{{\dot{Q}}_{CHE}}=\frac{T_{b}-T_{amb}}{V\;\cdot \;I}$$

**Figure 3:**
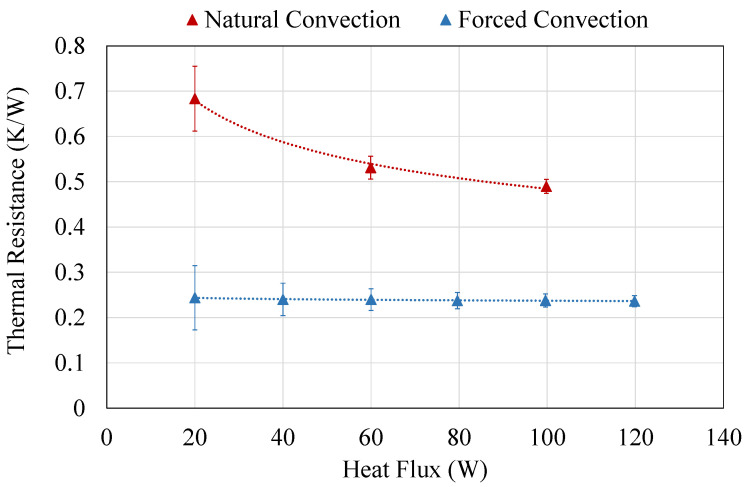
Thermal resistance of the cold side heat exchanger for different heat fluxes, considering natural and forced convection.

Figure 3 shows the thermal resistance of the heat exchanger with respect to the heat flux for the two conditions studied: natural and forced convection, the latter with a wind velocity of 4 m/s. As can be observed, in both cases the thermal resistance decreases as the heat flux increases. This occurs because the properties of the internal working fluid, which is cyclically experimenting phase change, improve with temperature, thus decreasing the thermal resistance for higher heat fluxes.

The former decrease is more noticeable for natural convection, where the thermal resistance diminishes from 0.68 K/W with 20 W, to 0.49 K/W with 100 W. In contrast, for forced convection the thermal resistance barely decreases 0.01 K/W from 20 to 120 W, presenting an average value of 0.24 K/W. The heat transfer coefficient in natural convection strongly depends on the temperature difference between the surface of the heat exchanger and the environment, improving with higher gradients. Nevertheless, in forced convection, this effect blurs in favor of the influence of wind velocity. Considering that this paper considers an outdoor application, the values of thermal resistance with forced convection are more representative.

**Figure 4:**
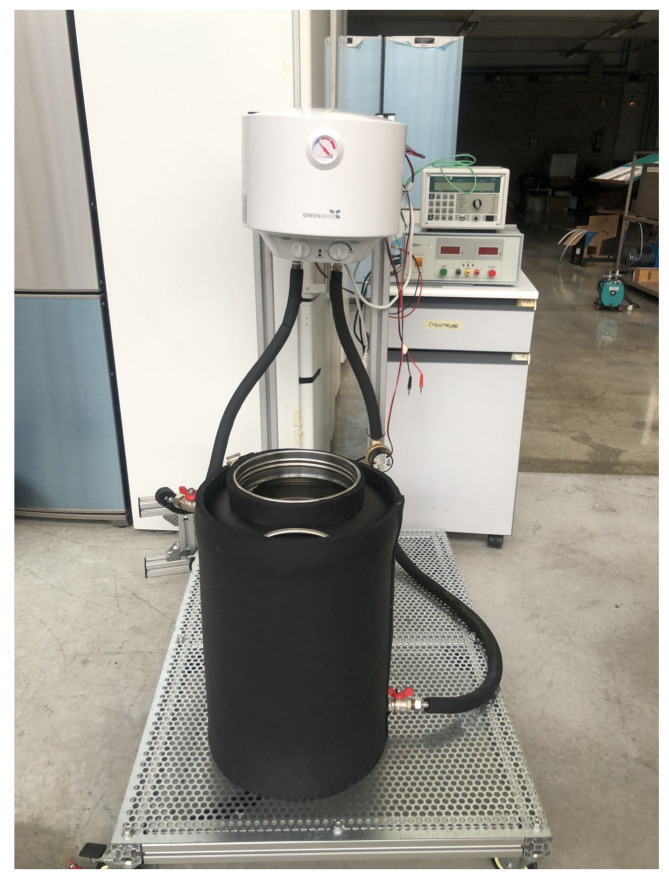
Thermal bath used as heat source in the laboratory experiments to determine the power generated by the designed prototype.

Apart from the characterization of the cold side heat exchanger, it is important to determine how much power can be generated with the designed prototype. At the laboratory, the experiments have been performed using the thermal bath depicted in Figure 4 as the heat source, while its analysis under real conditions is detailed in Section 6. This thermal bath is composed of a 10 L water heater, an insulated 30 L container, and a pump for the recirculation of the water used as the heat carrier. The designed prototype has been introduced in the insulated container, in direct contact with the water, which has been maintained at a practically constant temperature of 75 ${}^{{}^{\circ}}C$.

Utilizing the previous temperature constant heat source and with an ambient temperature of 16.5 ${}^{{}^{\circ}}C$, each thermoelectric module has been individually connected to an electrical load resistance and its voltage and intensity have been measured for the calculation of the power generated. More specifically, short-circuit, 1 Ω, 2.2 Ω, 3.2 Ω, 4.7 Ω and open-circuit conditions have been experimented and the uncertainties calculated again with [50]. In the experiments, the temperatures of the heat source $T_{source}$, the hot and the cold side of the thermoelectric modules $T_{H}$ and $T_{C}$, and the ambient temperature $T_{amb}$ have also been measured.

**Figure 5:**
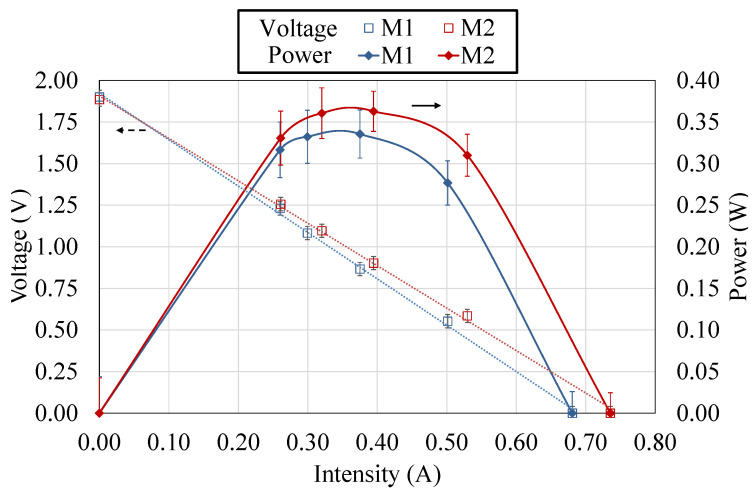
Voltage (left axis) and power generated (right axis) of the two thermoelectric modules studied, M1 and M2, with respect the intensity. The values correspond, from left to right, to open-circuit (OC), 4.7 Ω, 3.2 Ω, 2.2 Ω, 1 Ω and short-circuit (SC).

Figure 5 depicts, for the two thermoelectric modules M1 and M2, the voltage (left axis) and the power generated (right axis) with respect to the intensity. From left to right, the values correspond to open-circuit (OC), 4.7 Ω, 3.2 Ω, 2.2 Ω, 1 Ω and short-circuit (SC). As can be observed, the behavior of both thermoelectric modules is quite similar. As the load electrical resistance decreases, the intensity increases and the voltage decreases, with a linear relationship between them. In both cases, the open-circuit voltage is approximately 1.89 V while the short-circuit intensity presents a value of around 0.71 A (0.68 A M1, and 0.74 A M2).

Regarding power generation, depending on the electrical load resistance connected to the thermoelectric modules, generation varies, obtaining its maximum with a load resistance equal to the electrical internal resistance of the thermoelectric module. Hence, given an available temperature difference between sources of 58.81 ${}^{{}^{\circ}}C$ on average, module 1 (M1) generates a maximum of 0.34 W with a 2.2 Ω load resistance, which corresponds with a current of 0.38 A. On its behalf, module 2 (M2) presents a slightly higher maximum generation, 0.36 W, also with a load resistance of 2.2 Ω, that in this case corresponds with an intensity of 0.40 A. The small disparities between both thermoelectric modules are due to the different thermal contacts arisen from the assembly as well as to the differences in the heat exchangers, which were manually manufactured.

These disparities can be seen with more detail in Table 1. This table firstly displays, for each case, the temperatures of the heat source $T_{source}$, the hot and the cold side of the thermoelectric modules $T_{H}$ and $T_{C}$, and the ambient temperature $T_{amb}$. Based on the temperature difference of the cold side heat exchanger ($T_{C}-T_{amb}$) and its characterization, it is possible to make an iterative process and obtain the heat flux that is being released to the environment ${\dot{Q}}_{CHE}$ (Equation (1)). With this heat flux and the generated power *P*, from the energy balance shown in Equation (2) the heat absorbed from the heat source $\dot{Q}$ can be obtained, permitting the calculation of the efficiency of the thermoelectric modules (Equation (3)). Thus, it can be observed that the heat flux that goes through M2 is 4.75% higher than the one that goes through M1. This explains the higher generation of M2, which works with an efficiency of 1% with the optimal load resistance.

(2) $$\dot{Q}=P+{\dot{Q}}_{CHE}$$

(3) $$\eta =\frac{P}{\dot{Q}}$$

Thanks to the calculated heat flux and the measured temperatures, the thermal resistance of the hot side heat exchanger (HHE) has also been calculated (Equation (4)). Hence, the hot side heat exchanger of M1 presents a thermal resistance of 0.36 K/W on average, while the one of M2 is a bit smaller, 0.35 K/W. In comparison with the cold side heat exchanger (CHE), the value of which remains practically constant at 0.24 K/W, these resistances are 33 and 30.9% higher respectively. As a consequence, the temperature difference in the hot side heat exchangers is higher than in the cold ones (12.26 versus 8.15 ${}^{{}^{\circ}}C$ on average).

(4) $$R_{HHE}=\frac{T_{source}-T_{H}}{\dot{Q}}$$

## 5. Electronics

**Figure 6:**
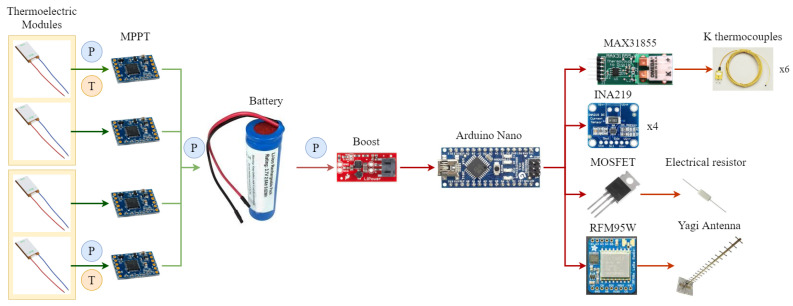
Diagram of the electronics installed with the prototype, which represents the node of the LoRa communication system.

The objective of the present paper is to develop an autonomous and robust volcanic monitoring station. Hence, since the generator part has already been analyzed, this section focuses on the electronic part associated to it prior to the installation of the whole system in a real volcano. These electronics, in order to resemble a real station, include a system to process the generated signal, as well as devices for acquiring data and emitting it wirelessly to a receptor. Their description will be based on the diagram shown in Figure 6, with the generation on the left side (with green arrows) and the consumption in the right one (with red ones). As can be observed, a prototype with four thermoelectric modules, this is two devices such as the one depicted in Figure 2, are considered to ensure a sufficient generation under real conditions, given that the heat transfer with the fumaroles is one of the greatest unknowns.

According to the previous section, it is important that the thermoelectric modules are connected to the optimal load electrical resistance. For this purpose, there exist some devices known as Maximum Power Point Trackers (MPPT) that perform this task. In thermoelectricity, the most common MPPT devices, which are also the simplest ones, adjust the output voltage of the thermoelectric modules to a value half of the open-circuit one [51]. In the present paper, each thermoelectric module has been individually connected to a Cypress MB39C831-EVB-03 Ultra Low Voltage Boost PMIC Energy Harvesting Evaluation Board [52], which works given an input voltage in the range between 0.3 and 4.75 V. As depicted in Figure 6, these boost converters are connected to a Li-ion battery. Hence, when the converter can obtain sufficient electric power from the thermoelectric modules, the charge is stared to the Li-ion battery until it reaches 4 V, when the charge is stopped. Charge restarts once the battery voltage has decreased to 3.7 V approximately. The RS Pro 18650 26H Li-ion battery pack has been used in this work [53].

On the demand side, data acquisition and communication systems are considered. In order to control them, it is necessary to include a microcontroller. In this case, Arduino Nano has been chosen because, presenting enough computational capacity, it has a very low energy consumption. Moreover, since all these components require a constant voltage of 5 V, which is higher than the battery’s one, a boost converter is required. This paper has installed a SparkFun PRT 10255 Boost Converter [54].

The monitoring system is in turn composed of two different parts. On the one hand, active sensing of six temperatures and four power generations is performed, thus simulating the existence of sensors in a real station while monitoring the operating conditions of the prototype. These measurements are taken every 4 min thanks to Maxim Integrated MAX31855PMB1 peripheral modules (with type K thermocouples) and Adafruit INA219 breakout boards respectively [55,56]. In order to avoid errors, these measurements are repeated 15 times, and the average of all the valid ones is stored. On the other hand, there is an extra consumption simulated passively with electrical resistors that includes peaks of demand, which are typical in vigilance stations. Hence, there is a constant power requirement of 0.3 W and every 12 min, a peak of consumption of 0.5 W occurs during 2 min. For this purpose, one of the outputs of the Arduino is connected to a MOSFET, so that an electrical resistor is directly connected to the battery when determined by the microcontroller.

All the data registered by the active sensors needs to be emitted to a reception center. Nevertheless, this process usually requires a high energy consumption [18]. Therefore, the present paper investigates low energy communication systems that, while being able to communicate wirelessly over a considerable distance, facilitate thermoelectric generation as the power supplier. Low-Power Wide-Area Network (LPWAN) standards, typically associated with Internet of Things (IoT), meet these requirements. Hence, this work analyzes the five technologies that seem to have the widest market potential: SigFox, LoRaWAN (Long Range WAN), IEEE 802.15 promoted by Wi-SUN (Wireless Smart Ubiquitous Network), and 3GPP standards: LTE-M (LTE for M2M) and NB-IoT (Narrow Band IoT) [57].

Table 2 summarizes the main characteristics of the former technologies in relation to their frequency band, maximum data rate, range (or coverage) and power usage [58,59,60,61,62,63]. As can be observed, within the table two categories can be distinguished. On the one hand, there is a category composed of LTE-M and NB-IoT, which use the current cellular telecommunications bands, since they are indeed extensions of the 4G network infrastructure. As a consequence, their range is extensive, but present a medium power usage, leading to their discard for the application under consideration in this paper.

On the other hand, SigFox, LoRaWAN, and Wi-SUN use the unlicensed ISM frequency bands and present low power usage. SigFox was one of the pioneers in the LPWAN market and it targets the very low power and low bandwidth applications, while offering very good coverage characteristics. Nevertheless, it is not available everywhere and it requires the payment of a royalty. On its behalf, LoRa is completely open and permits faster data rates, although the coverage range is curbed. Finally, Wi-SUN is focused on applications within public utilities, smart homes and smart cities, and therefore presents a reduced range of 5 km.

The present paper represents the first approximation for an autonomous volcanic monitoring station. Hence, the remoteness of volcanoes leads to the discard of Wi-SUN. Among SigFox and LoRa, the latter is preferred because, although it has a smaller coverage, it permits a higher data rate, it is completely free and it can be implemented everywhere. Due to these advantages, LoRa was also the technology chosen by Awadallah et al. and Terray et al. for their volcano surveillance applications, emitting the information up to 8.5 and 1.7 km, respectively [20,21].

**Figure 7:**
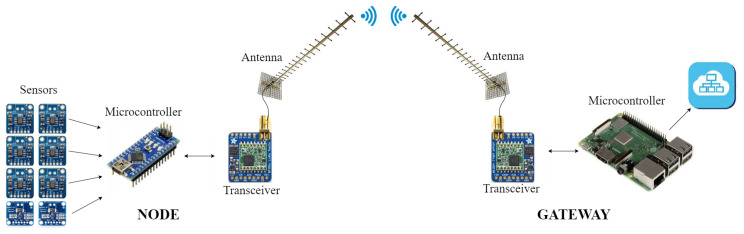
Schematics of a communication system implemented with LoRa.

Figure 7 details the complete solution provided by LoRa. Thus, the data acquired by the sensors is sent to a gateway located on a place with internet connection. For this purpose, it is necessary to install a transceiver and an antenna both at the node and at the gateway. In this paper, Adafruit RFM95W LoRa Radio Transceiver Breakout boards in conjunction with Yagi antennas have been used [64,65]. In the case of the gateway, a Raspberry Pi 3 B+ has been used as microcontroller, so that the received data is stored in an Influx database and synchronized with a server by means of MQTT protocol, so that the data can be represented with Grafana.

The power consumption in the node will depend on the frequency of emission, the distance, the size of the measured data, and the used sensors, transceiver, and microcontroller. Nonetheless, in order to have an approximation of the power requirement of such a system, a simplified experiment has been performed in this paper. Hence, a node composed of an Arduino Nano as microcontroller took, every 4 min, six temperature measurements with MAX31855 boards and two power measurements with Adafruit INA219s. The measured data was sent to a gateway located 13 km away by means of the mentioned Adafruit RFM95W board and Yagi antenna. As a result, the power demand in the node was 0.11 W on average, reaching peaks of 0.246 W during the emission. Therefore, LoRa is definitely chosen as the communication system in this paper, since it is able to emit wirelessly the measured data over a considerable distance while presenting a minimal energy consumption, thus facilitating thermoelectric generation as the power supplier.

The emission of data from the prototype node to the gateway has been programmed to be bi-directional and encrypted. Thus, the prototype emits, every four minutes, the measured data and waits for the confirmation of reception of the message. This process is performed with an initial spreading factor of nine. If the confirmation is not received after 60 s, a second attempt starts. In case neither confirmation is received, a third and last attempt is performed with a spreading factor of 10, so that the range is increased. If the confirmation from the gateway is received, the prototype node sends a new message to the gateway and both nodes update their encrypted key.

**Figure 8:**
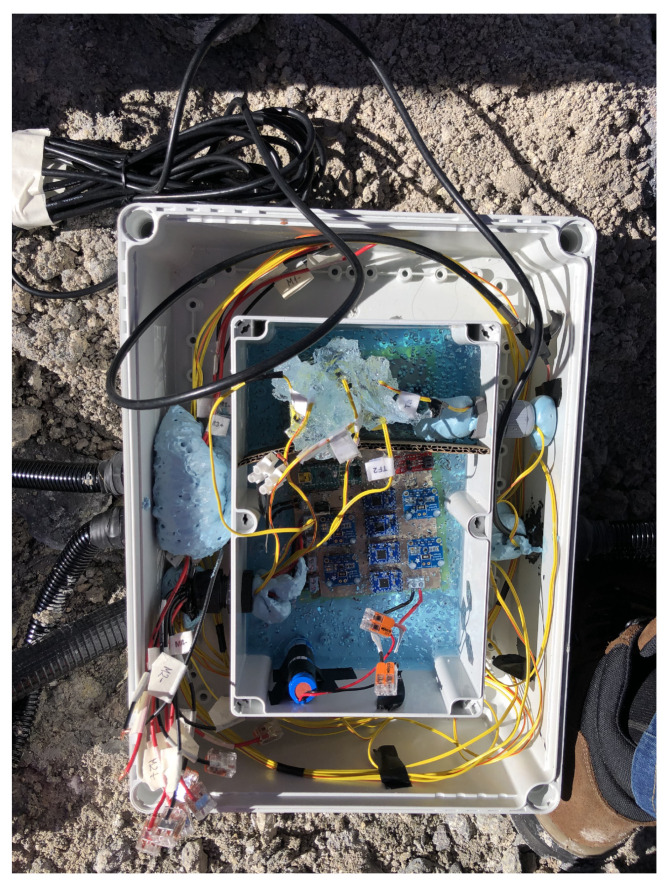
Detail of the protection boxes to avoid corrosion in the PCB.

Finally, similarly to the prototype, and due to the harsh volcanic environmental conditions, it is necessary to protect the electronics to stand severe conditions. Figure 8 depicts the protection measures that have been taken. Thus, the PCB with all the electronics has been introduced in an IP67 plastic box and covered with Raytech magic power gel [66]. This box has been closed and introduced in another IP67 plastic box. Since the different cables need to be taken out of the boxes, cable glands have been installed with a Nylon conduit contractor, and the connections have been protected with self-amalgamating tape and polyurethane foam.

## 6. Behavior on a Real Volcano

**Figure 9:**
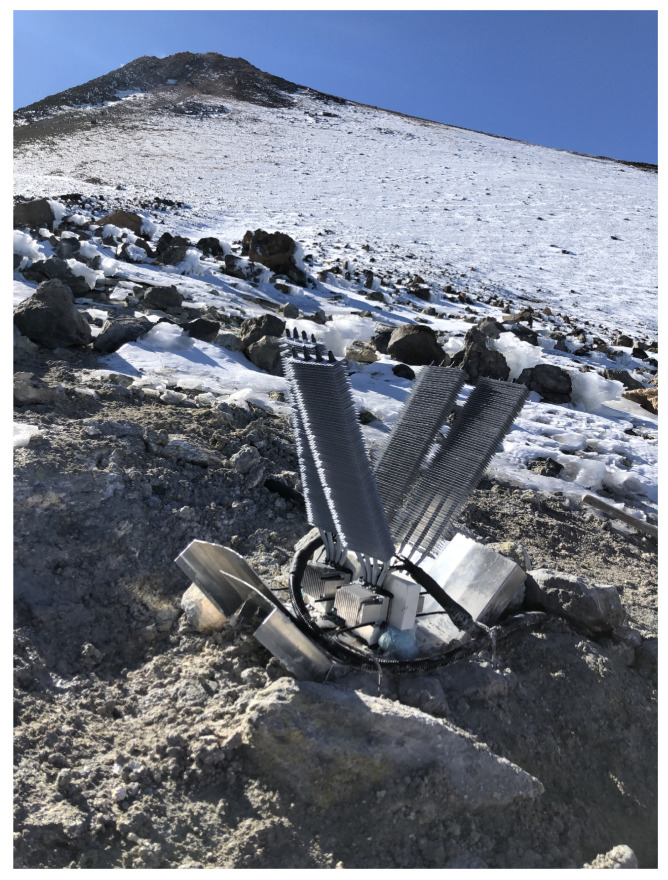
Prototype installed at Teide volcano on December 2019, composed of 4 thermoelectric modules.

**Figure 10:**
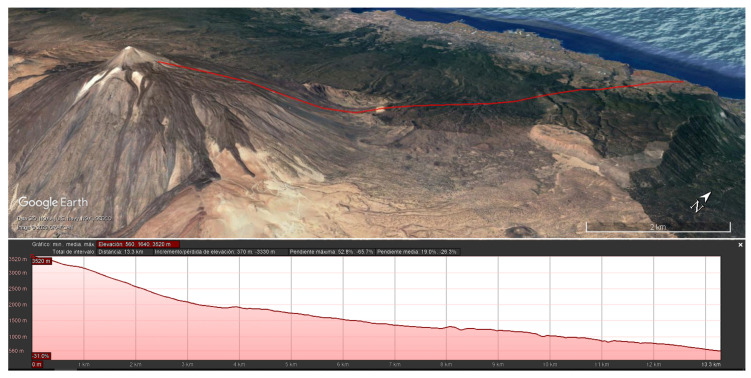
Location of the prototype node and the gateway, and their associated elevation profile. Images taken from Google Earth ©.

On 18th December 2019, the prototype was installed at Teide volcano (Canary Islands, Spain) with its associated electronics (Figure 9) and on the submission date of this manuscript, the prototype was still in operation. Teide is a stratovolcano located in the Canary Islands (Spain), an archipelago of volcanic origin that emerges in the Atlantic Ocean. With an altitude of 3718 m, Teide is one of the most evident manifestations of the active volcanism of the islands, and therefore, it is widely monitored. Figure 10 details the location of the prototype, in the northern side of the volcano at an approximate altitude of 3500 m, as well as the position of the gateway, 14 k m away with direct vision. As shown in Figure 9, in its installation, aluminum deflectors were added so that the volcanic gases are diverted, not coming into contact with the thermoelectric modules nor the cold side heat exchangers. Polyurethane foam was used for sealing the joints. Furthermore, polyethylene was also added in order to insulate the aluminum block of the hot side heat exchanger and the thermoelectric modules from the influence of ambient conditions.

**Figure 11:**
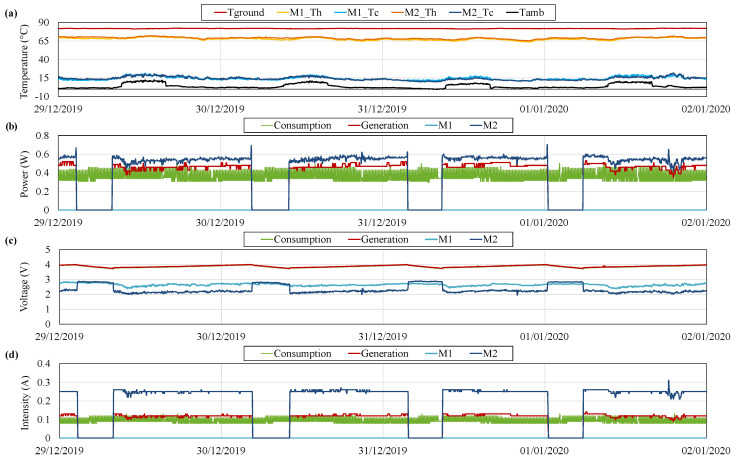
(**a**) Temperature of the ground *T_ground_*, the hot and the cold side of two thermoelectric modules M1 and M2 (*T_H_* and *T_C_*) and the ambient *T_amb_*, (**b**) power, (**c**) voltage and (**d**) intensity measurements during typical operation.

Figure 11 depicts the typical operation of the prototype, taking as reference the variables measured between 29 December 2019 and 2 January 2020. More specifically, Figure 11a shows the temperature of the ground $T_{ground}$, the hot and the cold side of two thermoelectric modules $M1\_T_{H}$, $M1\_T_{C}$, $M2\_T_{H}$ and $M2\_T_{C}$, and the ambient temperature $T_{amb}$. Each monitored thermoelectric module belongs to one of the individual generators installed.

As can be observed, ground temperature $T_{ground}$ remains practically constant with an average value of 81.82 ${}^{{}^{\circ}}C$. At the opposite side of the prototype, the ambient temperature $T_{amb}$ presents the typical fluctuations of day and night. Hence, since the considered values correspond to winter, during the night, temperatures close to 0 ${}^{{}^{\circ}}C$ are found, while during the day, the temperatures rise up to an average of 10 ${}^{{}^{\circ}}C$.

The difference between the ground and the ambient temperatures ($T_{ground}-T_{amb}$) represents the maximum temperature difference achievable by the thermoelectric modules, although, in reality, the temperature difference between the hot and the cold side of the modules will be lower. The approximation of the temperature difference of the thermoelectric modules ($T_{H}-T_{C}$) to the maximum available one ($T_{ground}-T_{amb}$) depends on the heat exchangers installed. The lower the thermal resistances of the heat exchangers, the closer the temperature differences.

In the considered hot side heat exchangers, there exists an average temperature difference of 13.5 ${}^{{}^{\circ}}C$, while this difference is of 10.5 ${}^{{}^{\circ}}C$ in the case of the cold side ones. Thus, the thermoelectric modules work under a gradient of 54 ${}^{{}^{\circ}}C$, which is similar for the two thermoelectric modules that are being monitored. This gradient is slightly influenced by the ambient conditions, decreasing during the day and increasing during the night, which will affect generation.

Figure 11b–d show the values associated with the measurements performed by means of the INA219s: power, voltage, and intensity respectively. In particular, these sensors, as shown in Figure 6, have been installed monitoring the individual power generated by the thermoelectric modules M1 and M2 of which the temperatures are also being measured (before the MPPT), the generation of all the thermoelectric modules (after the MPPTs and before the battery), and the consumption of the different devices (at the battery output).

As explained before, the consumption profile presents a cyclic oscillation, with a fixed power requirement of 0.3 W and peaks of 0.5 W during 120 s every 12 min. Since the measurement of the different variables is performed every four minutes, the consumption profile is shown as peaky rather than pulsed, as can be observed in green in Figure 11b. The measurement of this consumption is being performed at the output of the battery, or in other words, at the input of the PRT boost converter that adapts the battery voltage to 5 V, so that the consumption of all the charges is considered. Thus, the voltage of the consumption coincides with the battery one in Figure 11c.

In order to satisfy the previous demand, four thermoelectric modules were installed. Nevertheless, the generation supplied by only one module is enough for this purpose. As can be observed in Figure 11b, the generation of the four thermoelectric modules, in red, is slightly lower than the generation of M2, in dark blue, while the generation of M1, in light blue, is null. This means that out of the four thermoelectric modules installed, only M2 is in operation. The generation of the individual modules is measured before the MPPT. Therefore, the difference between the total generation and the individual generation of M2 is due to the conversion efficiency of the MPPT, which can be rated as 85.7% in this case.

The total generation is around 0.47 W, higher than the consumption, which presents an average value of 0.37 W. Hence, the battery is charged with the excess of energy produced, until it reaches 4 V (Figure 11c). At this moment, the MPPT opens the circuit of M2 for security reasons, leading to a null intensity and increasing the voltage of M2 from 2.18 to 2.82 V approximately. This open-circuit value is very similar to the one displayed by M1.

When all the modules are in open-circuit, the battery is progressively discharged, reducing its voltage. When this voltage reaches 3.74 V, M2 activates again, starting a new cycle. The threshold at which the thermoelectric modules activate is determined by the MPPT. Nonetheless, there exist slight differences in the used boards. M2 has the highest threshold and therefore, it is the first one that starts its operation. It would be necessary to have a battery voltage lower than 3.74 V for the activation of other thermoelectric modules.

**Figure 12:**
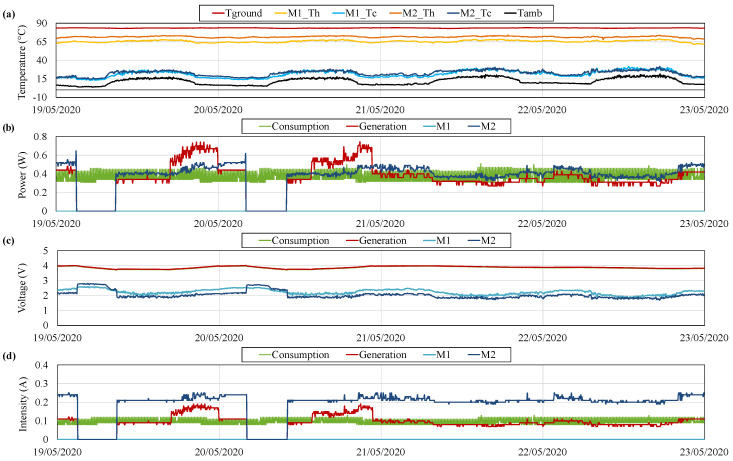
(**a**) Temperature, (**b**) power, (**c**) voltage and (**d**) intensity measurements during several hot days.

If the ambient temperature increases, the available temperature difference decreases, as so does the gradient between the sides of the thermoelectric modules, and therefore, the generation. Figure 12 depicts the variables measured between 19 and 23 May 2020, when temperatures close to the typical maximums at Teide volcano were recorded [67]. In this period, the ambient temperature presents values of around 8 ${}^{{}^{\circ}}C$ during the night and up to 22 ${}^{{}^{\circ}}C$ during the day. Consequently, the temperature difference between the sides of the modules has decreased from the previous case, with an average of 49 ${}^{{}^{\circ}}C$.

**Figure 13:**
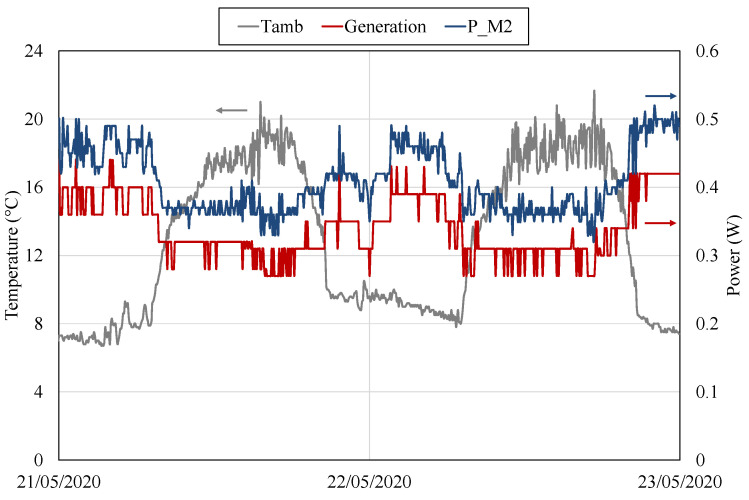
Ambient temperature $T_{amb}$ (left axis), power generated by M2 and total generation (right axis) between 21 and 23 May 2020.

As shown in Figure 12, the total generation obtained with the former gradient is of just 0.35 W, lower than the average consumption. Therefore, the battery is gradually discharged although one thermoelectric module is in operation. When the voltage of the battery is lower than 3.73 V, another thermoelectric module starts its operation. The individual generation of this module is not being monitored, but it can be seen that the total generation, in red, exceeds the individual generation of M2, while M1 remains in open-circuit. When the battery voltage reaches 3.99 V, this second thermoelectric module switches to open-circuit, and again M2 is the only module in operation. On 20 May, the total generation with only M2 was higher than the consumption, completely charging the battery a couple of hours after the disconnection of the second module. Nevertheless, on the 21, when the second module switched to open-circuit, total generation with M2 was similar to the consumption. Hence, an equilibrium between generation and consumption can be observed, discharging the battery during the day and charging it during the night, but without reaching the upper nor the lower thresholds to switch to open-circuit or force the operation of a new module respectively. During this equilibrium period, the influence of ambient temperature in generation can be perfectly seen. This influence is more detailed in Figure 13, which shows the ambient temperature $T_{amb}$ (left axis), the total power generation and the power generated by M2 (right axis) between 21 and 23 May 2020. Thus, during the day temperature increases, diminishing the available temperature difference and therefore reducing generation. During the night, this temperature decreases and generation increases.

The ambient temperature during the days depicted in Figure 12 is very similar to the one used in the laboratory characterizations. Hence, assuming that the wind conditions at Teide are similar to the laboratory ones (in forced convection), an estimation of the heat flux, the thermal resistance of the heat exchangers, and the efficiency can be performed.

Table 3 summarizes the average results of M2 on 19 May between 11:00 and 20:00 h. As can be observed, at Teide, ground temperature is almost 10 ${}^{{}^{\circ}}C$ higher than the laboratory’s heat source’s one. Consequently, the heat flux increases, reaching 40 W in this case and raising the hot side temperature to 72.5 ${}^{{}^{\circ}}C$. Since the gradient between the sides of the module is higher, so is generation, with an average value of 0.41 W, which entails an efficiency of 1.02%.

Based on the previous data, it is also possible to calculate the thermal resistance of the hot side heat exchanger. Hence, with Equation (4) a value of 0.27 K/W is obtained, 23% lower than at the laboratory, where natural convection with water was the heat transfer mechanism. Heat transfer between the ground and the hot side heat exchanger was one of the greatest unknowns, complicating its simulation at the laboratory. Nonetheless, it has been experimentally demonstrated that this heat transfer is really acceptable, with a thermal resistance quite similar to the cold side one. The fumaroles heat up and moisten the ground in their ascent to the surface. Thus, heat transfer is not produced by convection with the gaseous fumaroles but by conduction with the hot and wet ground, which permits obtaining better heat transfer coefficients.

**Figure 14:**
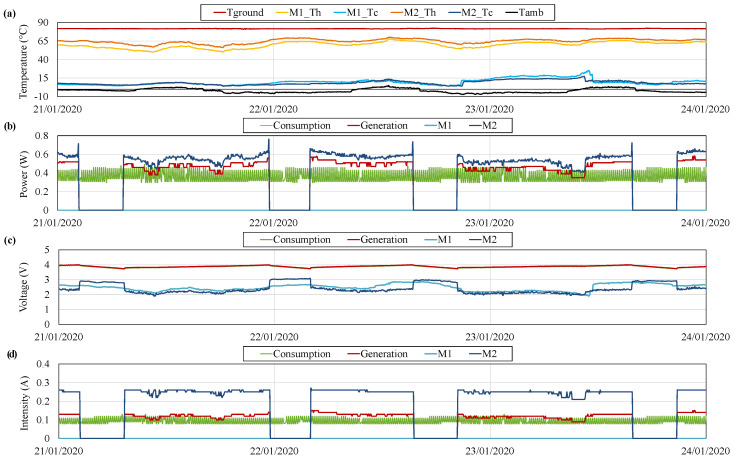
(**a**) Temperature, (**b**) power, (**c**) voltage and (**d**) intensity measurements during several cold days.

Since its installation in December 2019, the prototype has undergone multiple kinds of meteorological conditions, from severe snowfalls and frosts to days with the typical Saharan air layer. On the one hand, Figure 14 shows its operation between 21 and 24 January 2020, when ambient temperature reached values down to −10 ${}^{{}^{\circ}}C$. As can be seen, due to the greater difference between ground and ambient temperature, the gradient between the sides of the thermoelectric modules increases, also raising the generation, which reaches values of up to 0.66 W in the case of M2. Despite its operation with temperatures below 0 ${}^{{}^{\circ}}C$, the cold side heat exchanger does not present signs of freezing, except a very punctual moment. The night between 22 and 23 January, an uncoupling between the ambient and the modules’ cold side temperature can be observed, which could be due to the freezing of the cold side heat exchangers. Nevertheless, this fact only causes a slight reduction in the power generated, which is not even enough to reduce the total generation below the demand.

On the other hand, the prototype has also dealt with other extreme meteorological conditions such as the typical Saharan air layer. This phenomenon, also known as *Calima* in the Canary Islands, is characterized by a hot, dry, and dust-laden atmosphere. As shown before, high temperatures are not a problem for the operation of the prototype. The most critical aspect of this phenomenon is the reduced visibility caused by dust plumes, which could affect the communication with the gateway, located 14 k m away. Nevertheless, since its installation, there have not been data losses. Not even in February, when one of the worst *Calima* episodes in the last 30 years affected the Canary Islands [68].

The visibility loss can also occur due to the formation of clouds between the emitter and the gateway. At Teide, it is common to observe a temperature inversion that leads to the so-called *sea of clouds* due to its appearance from above. Nevertheless, despite occurring quite often, this phenomenon neither affects the communication system. Thus, the prototype, and its associated electronics, have demonstrated to withstand several types of meteorological conditions, being able to supply the required energy mostly with only one thermoelectric module and ensuring communication at every moment.

Table 4 summarizes the number of hours that each thermoelectric module has been in operation between 19 December 2019 and 20 August 2020. As can be observed, M2 is the thermoelectric module that leads generation, with more than 4400 h of operation. M3 is the other module that supplies power occasionally, with less than 270 h being active, while M1 and M4 are never necessary. On average, M2 is in operation 86.2% of the time, being in open-circuit the rest of the time. Nonetheless, depending on the ambient conditions, this percentage of time in operation can vary. In the table, apart from the average values, it is also represented by the percentage under the typical conditions shown in Figure 11, under the hot days depicted in Figure 12, and under the cold days considered in Figure 14. Hence, when the ambient temperature decreases, due to the higher generation of the thermoelectric modules, M2 needs to be active a shorter period of time, while in hot days, the operation time increases. It is in these hot days when it is sometimes required that another thermoelectric module, M3, starts its operation, when the generation of M2 is lower than the consumption and the battery is being discharged. Nevertheless, the rest of the time is in open circuit, leading to an average of 0.9% of the time in operation. Thus, it can be seen that during 93.95% of the time that the prototype has been installed at Teide volcano, the generation of M2 has been enough to supply the required power.

**Figure 15:**
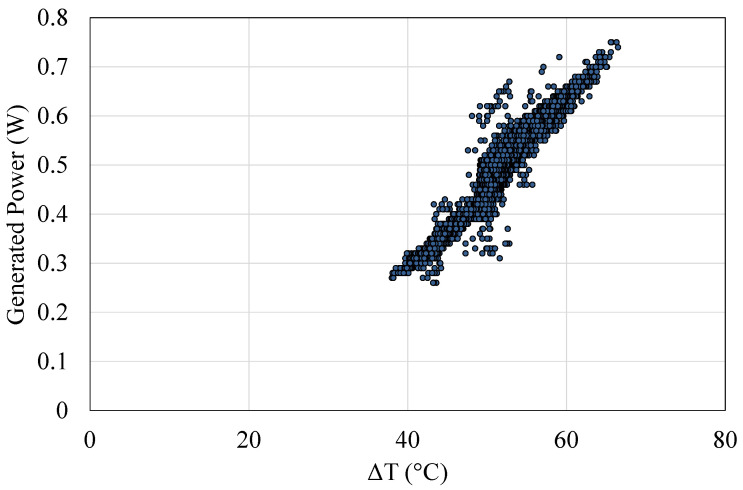
Power generated by M2 versus the temperature difference between their sides $\Delta T=T_{H}-T_{C}$ between 19 December 2019 and 20 August.

Finally, Figure 15 shows the power generated by M2 with respect to the temperature difference between its sides $\Delta T=T_{H}-T_{C}$ during the previous period. As can be observed, there exists a linear correlation between the generated power and the temperature difference between its sides. This correlation has remained unaltered during the eight months analyzed, presenting an average value of 0.49 W with a 51.5 ${}^{{}^{\circ}}C$ gradient that supposes 2.2 kWh of energy produced. Therefore, the robustness and the resistance of the developed prototype to the harsh volcanic conditions has been evidenced, even more taking into account that no maintenance has been carried out.

## 7. Supplying Power to a Real Vigilance Station

In this last section the viability of supplying power to a real station is analyzed. Clearly, depending on the station under consideration, with its associated equipment, a different power supply will be necessary. Nonetheless, since one of the main advantages of thermoelectricity is its scalability, an increase in the generated power can be simply achieved by installing more thermoelectric modules (with their associated heat exchangers).

This section considers as reference one of the vigilance stations located at Teide volcano. More specifically, the one located at “La Fortaleza” lookout, which is next to the installed prototype and belongs to the Volcanological Institute of the Canary Islands [69]. The station is composed of a seismograph, a WEST continuous monitoring flux unit that measures the concentration of several gases (CO${}_{2}$, H${}_{2}$S, CH${}_{4}$), soil moisture and temperature, as well as various meteorological parameters [70], and a GSM router to emit the measured data to the control center.

The power consumption of the station was monitored over two days. As a result, it was obtained that both the seismograph and the router present a practically uniform power consumption of 1.86 W and 3.77 W on average respectively. In contrast, the flux unit presents an average demand of 1.5 W that is not constant but with peaks of demand once an hour.

Nowadays, this consumption is powered by means of a 175 W photovoltaic panel of dimensions 1306 × 991 × 40 m m ${}^{3}$. This panel is connected to a Victron BlueSolar MPPT 75∣15 [71], a charge controller that maximizes energy harvesting from the panel and stores it in a 1260 Wh lead-acid battery. The system is over-dimensioned in order to ensure the vigilance of the volcano despite snow periods up to 3.7 days. Nevertheless, it complicates maintenance due to its size and weight.

The solution proposed in the present paper, based on a geothermal thermoelectric generator, permits a more uniform generation that even improves with adverse meteorological conditions. Therefore, it would be possible to reduce the energy storage requirements or replace it with other technologies such as supercapacitors. Nonetheless, in order to make it possible, it is desirable to reduce the power demand to a minimum due to the low efficiency of the thermoelectric modules. In the power measurements, the high power demand of the router stands out, which is two and three times the average consumption of the seismograph and the flux unit respectively, while being the least critical for the volcano vigilance and the easiest one to be modified.

As explained in Section 5, LoRa is able to communicate wirelessly over a considerable distance with a minimal consumption of just 0.11 W, which supposes a reduction of 97% in the power requirement of the communication system, leading to a reduced number of thermoelectric modules necessary in order to obtain a completely autonomous volcanic monitoring station. More specifically, taking into account all the previous information, in order to supply an average power of 3.24 W, seven thermoelectric modules would be necessary, that is, two devices such as the one depicted in Figure 9. Therefore, the solution proposed in this paper will be perfectly viable to power a real volcanic monitoring station.

## 8. Conclusions

In conclusion, the present paper has demonstrated the viability of obtaining autonomous volcanic vigilance stations. Volcano surveillance is essential in order to predict volcanic eruptions and be able to reduce their damage. However, due to the usual remoteness of volcanoes, power supply generally constitutes a challenge.

As a solution, this work has developed a novel system composed of thermoelectric generators as power suppliers, as well as all their related electronics, for which technologies typically associated with Internet of Things (IoT) have been used. Thus, thanks to the heat emitted in volcanic fumaroles, which is indeed a sign of activity of the volcanoes, electricity can be directly generated by means of the Seebeck effect. Since fumaroles are a constant temperature heat source, a continuous generation is obtained regardless of the weather conditions, permitting a drastic reduction in the capacity of the required batteries. Thanks to this generation, it is possible to measure different variables and emit the results via LoRa to a gateway located several kilometers away.

In order to analyze the viability of this solution, a prototype formed by heat pipes as heat exchangers has been characterized at the laboratory and installed afterward at Teide volcano (Canary Islands, Spain), where there exists 83.5 ${}^{{}^{\circ}}C$ fumaroles. In more than eight months of operation, the device has demonstrated that only one module is enough to cover the demand of data acquisition and communication over 14 km. On average, this thermoelectric module has generated 0.49 W with a temperature difference of 51.5 ${}^{{}^{\circ}}C$.

During its operation, the prototype has resisted, without any maintenance, several kinds of adverse meteorological conditions in a very harsh environment. Therefore, the viability of the solution has been demonstrated, evidencing all its advantages: durability, reliability, lack of maintenance, scalability, absence of moving parts, noiseless operation, robustness, and compactness.

## 9. Patents

The mode of operation of the developed thermoelectric generator is patented under number WO 2019/202180 A1.

## Figures and Tables

**Table 1:** For each thermoelectric module (M1 and M2) and load electrical resistance $R_{load}$, temperatures of the heat source $T_{source}$, the hot and cold side of the thermoelectric modules $T_{H}$ and $T_{C}$, and ambient $T_{amb}$, power generated *P*, heat flux extracted from the source $\dot{Q}$, efficiency of the thermoelectric modules η, and thermal resistances of the hot and cold side heat exchangers (HHE and CHE respectively).

Thermoelectric	$R_{\mathrm{load}}$	$T_{\mathrm{source}}$	$T_{H}$	$T_{C}$	$T_{\mathrm{amb}}$	*P*	$\dot{Q}$	η	$R_{\mathrm{HHE}}$	$R_{\mathrm{CHE}}$
Module	(Ω)	( ${}^{{}^{\circ}}$C)	( ${}^{{}^{\circ}}$C)	( ${}^{{}^{\circ}}$C)	( ${}^{{}^{\circ}}$C)	(W)	(W)	(%)	(K/W)	(K/W)
M1	SC	74.23	61.90	25.47	16.53	0.00	37.01	0.00%	0.33	0.24
	1	76.23	62.50	24.96	16.42	0.28	35.34	0.78%	0.39	0.24
	2.2	75.08	62.75	24.85	16.57	0.34	34.22	0.97%	0.36	0.24
	3.2	75.40	63.19	24.64	16.56	0.33	33.38	0.99%	0.36	0.24
	4.7	75.92	63.73	24.51	16.44	0.32	33.36	0.94%	0.36	0.24
	OC	74.82	64.01	23.76	16.31	0.00	30.75	0.00%	0.35	0.24
M2	SC	74.23	61.53	26.11	16.53	0.00	39.72	0.00%	0.32	0.24
	1	76.23	62.32	25.37	16.42	0.31	37.07	0.83%	0.37	0.24
	2.2	75.08	62.51	25.21	16.57	0.36	35.76	1.00%	0.35	0.24
	3.2	75.40	63.02	25.01	16.56	0.36	34.96	1.02%	0.35	0.24
	4.7	75.92	63.59	24.84	16.44	0.33	34.77	0.94%	0.35	0.24
	OC	74.82	63.84	24.05	16.31	0.00	31.95	0.00%	0.34	0.24

**Table 2:** Comparison of some Low-Power Wide-Area Network (LPWAN) technologies [58,59,60,61,62,63].

Technology	Frequency Band	Maximum Data Rate	Range	Power Usage
SigFox	Sub-GHz ISM	600 bbps	10 km (urban), 50 km (rural)	Low
LoRaWAN	Sub-GHz ISM	50 kbps	5 km (urban), 15 km (rural)	Low
Wi-SUN	Sub-Ghz ISM 2.4 GHz	300 kbps	5 km	Low
LTE-M	Cellular Bands	1 Mbps	Several km	Medium
NB-IoT	Cellular Bands	100 kbps	Several km	Medium

**Table 3:** Average temperatures, generation and thermal resistances of M2 during 19 May 2020 between 11:00 and 20:00 h.

Temperatures		Generation		Thermal Resistances	
$T_{ground}$	83.44 ${}^{{}^{\circ}}C$	*P*	0.41 W	$R_{HHE}$	0.27K/W
$T_{H}$	72.53 ${}^{{}^{\circ}}C$	$\dot{Q}$	40.00 W	$R_{CHE}$	0.24K/W
$T_{C}$	24.77 ${}^{{}^{\circ}}C$	η	1.02%		
$T_{amb}$	15.12 ${}^{{}^{\circ}}C$				

**Table 4:** Total hours in operation and percentage of time active of the four thermoelectric modules installed, considering the average between 19 December 2019 and 20 August 2020, as well as their behavior under typical conditions (Figure 11), hot days (Figure 12) and cold days (Figure 14).

Thermoelectric	Total Time	Percentage of Time in Operation (%)			
Module	in Operation (h)	Average	Typical	Hot Days	Cold Days
M1	0	0%	0%	0%	0%
M2	4441.4	86.2%	78.2%	87.6%	73.4%
M3	269.1	5.2%	0%	16.8%	0%
M4	0	0%	0%	0%	0%

## Abbreviations

The following abbreviations are used in this manuscript:

CHE	Cold side heat exchanger
GTEG	Geothermal Thermoelectric Generator
HHE	Hot side heat exchanger
IoT	Internet of Things
LPWAN	Low Power Wide Area Network
MPPT	Maximum Power Point Tracker
M1	Thermoelectric module 1
M2	Thermoelectric module 2
OC	Open-circuit
SC	Short-circuit
*I*	Intensity (A)
*P*	Power (W)
$\dot{Q}$	Heat flux (W)
*R*	Thermal resistance (K/W)
$R_{load}$	Load electrical resistance (Ω)
*T*	Temperature (${}^{{}^{\circ}}C$)
*V*	Voltage (V)
η	Efficiency (%)
amb	Ambient
*b*	Base of the heat exchanger
*C*	Cold side of the thermoelectric module
*H*	Hot side of the thermoelectric module
source	Heat source
